# The Assessment of Supportive Accountability in Adults Seeking Obesity Treatment: Psychometric Validation Study

**DOI:** 10.2196/17967

**Published:** 2020-07-28

**Authors:** Karishma Chhabria, Kathryn M Ross, Shane J Sacco, Tricia M Leahey

**Affiliations:** 1 The Center for Health Care Data Department of Management, Policy and Community Health University of Texas Health Science Center at Houston Houston, TX United States; 2 Department of Clinical and Health Psychology University of Florida Gainesville, FL United States; 3 Department of Allied Health Sciences University of Connecticut Storrs, CT United States

**Keywords:** supportive accountability, social support, SALLIS, factor analysis, obesity, weight loss, technology, mobile phone

## Abstract

**Background:**

Technology-mediated obesity treatments are commonly affected by poor long-term adherence. Supportive Accountability Theory suggests that the provision of social support and oversight toward goals may help to maintain adherence in technology-mediated treatments. However, no tool exists to measure the construct of supportive accountability.

**Objective:**

This study aimed to develop and psychometrically validate a supportive accountability measure (SAM) by examining its performance in technology-mediated obesity treatment.

**Methods:**

Secondary data analyses were conducted in 2 obesity treatment studies to validate the SAM (20 items). Study 1 examined reliability, criterion validity, and construct validity using an exploratory factor analysis in individuals seeking obesity treatment. Study 2 examined the construct validity of SAM in technology-mediated interventions involving different self-monitoring tools and varying amounts of phone-based interventionist support. Participants received traditional self-monitoring tools (standard, in-home self-monitoring scale [SC group]), newer, technology-based self-monitoring tools (TECH group), or these newer technology tools plus additional phone-based support (TECH+PHONE group). Given that the TECH+PHONE group involves more interventionist support, we hypothesized that this group would have greater supportive accountability than the other 2 arms.

**Results:**

In Study 1 (n=353), the SAM showed strong reliability (Cronbach α=.92). A factor analysis revealed a 3-factor solution (representing Support for Healthy Eating Habits, Support for Exercise Habits, and Perceptions of Accountability) that explained 69% of the variance. Convergent validity was established using items from the motivation for weight loss scale, specifically the social regulation subscale (r=0.33; *P*<.001) and social pressure for weight loss subscale (r=0.23; *P*<.001). In Study 2 (n=80), the TECH+PHONE group reported significantly higher SAM scores at 6 months compared with the SC and TECH groups (r^2^=0.45; *P*<.001). Higher SAM scores were associated with higher adherence to weight management behaviors, including higher scores on subscales representing healthy dietary choices, the use of self-monitoring strategies, and positive psychological coping with weight management challenges. The association between total SAM scores and percent weight change was in the expected direction but not statistically significant (r=−0.26; *P*=.06).

**Conclusions:**

The SAM has strong reliability and validity across the 2 studies. Future studies may consider using the SAM in technology-mediated weight loss treatment to better understand whether support and accountability are adequately represented and how supportive accountability impacts treatment adherence and outcomes.

**Trial Registration:**

ClinicalTrials.gov NCT01999244; https://clinicaltrials.gov/ct2/show/NCT01999244

## Introduction

### Background

Technology-mediated health behavior change interventions have become ubiquitous [1-4]. Adherence tends to drop off dramatically over time, such that few participants meet the program goals related to the use of technology-based intervention tools (eg, self-monitoring platforms) by the end of treatment [5-7]. Importantly, this poor adherence may lead to suboptimal treatment outcomes. Although technology-based interventions can lead to clinically significant weight loss in some participants [8], most of these programs demonstrate subpar outcomes in relation to in-person treatments [9,10]. For example, electronically delivered behavioral weight loss programs commonly achieve a weight loss of 3 kg to 5 kg within 3 to 6 months of treatment [11,12], whereas traditional in-person programs often produce a weight loss of 8 kg to 10 kg within the same time frame [11,13].

Given this challenge, research has focused on methods to improve adherence to technology-based behavioral interventions. To date, one of the most effective strategies has been the provision of additional support (eg, via telephone, email, text messages or via smartphone apps) from interventionists or lifestyle coaches [14-16]. Indeed, existing guidelines for the treatment of overweight and obesity specifically note that programs delivered electronically should provide personalized feedback from an interventionist and that primarily knowledge-based internet programs may be ineffective [11].

Consistent with these empirical findings, Mohr et al [17] recently proposed Supportive Accountability Theory, which posits that adherence to technology-based interventions may be improved through the provision of both social support (whether delivered in person or electronically and either synchronously or asynchronously) and accountability (the expectation that an individual would regularly have to explain their progress toward program goals). Despite the utility of Supportive Accountability Theory for identifying important potential mechanisms for improving adherence and, ultimately, program outcomes in technology-based interventions, there is no validated measure to assess supportive accountability.

### Objectives

The objective of this study was to describe the development and validation of a measure for assessing supportive accountability within the context of technology-mediated programs for the treatment of adult obesity. Building off of the Social Support for Diet and Exercise Behaviors Scale by Sallis et al [18], a widely used measure of social support for healthy eating and activity, new items were added to represent the construct of accountability as described by Mohr et al [17]. The psychometric characteristics of this new measure (Supportive Accountability Measure [SAM]) were assessed using data collected from 2 weight loss trials. Baseline data from the first trial (Study 1) aimed to examine reliability, conduct an exploratory factor analysis (EFA), and assess convergent validity. Data from the second trial (Study 2) aimed to further investigate the criterion validity of the new measure. Specifically, Study 2 examined the impact of newer self-monitoring technology and phone coaching on weight loss; thus, it was hypothesized that participants who were randomized to receive additional phone coaching, in addition to technology-based self-monitoring tools, would report higher supportive accountability compared with participants not provided with this additional coaching.

## Methods

### Supportive Accountability Measure Development

The SAM was developed by adding novel items representing the construct of accountability to items from the existing Social Support for Diet and Exercise Behaviors Scale developed by Sallis et al [18], a widely used social support measure in the weight management literature [15]. First, questions assessing accountability were developed by a senior author (TL), drawing on Mohr’s description of Supportive Accountability Theory [17] and her expertise in the field of obesity treatment. Items were developed to assess accountability for typical behavioral intervention components, including goals related to weight loss, dietary intake, and physical activity. Participants respond to items on this scale using a 7-point Likert scale, with response options ranging from 1 (*not at all*) to 7 (*very much*). A total of 10 items were judged to fully capture all facets of this construct (supporting content validity) and were thus retained. Multimedia Appendix 1 shows the final accountability items selected for the SAM.

To ensure balance in the number of items, 10 items were selected from the Sallis Social Support measure [17]. The original Sallis measure includes 43 items; some assess social support, whereas others assess sabotage, negative comments, and punishment. Overall, these subscales have demonstrated good internal consistency, criterion validity, and construct validity and acceptable test-retest reliability [18]; however, less support has been found for the utility of the negative subscales (Negative Comments, Sabotage, and Punishment) [18,19]. Given the lack of psychometric support for the negative subscales and that the questions focused on negative comments and sabotage did not align with the theoretical construct of supportive accountability, the 10 items that were retained from the Sallis measure were those that assessed positive support for healthy eating and exercise [18]. Participants were asked to rate support received from family, friends, and their weight loss coach on the 10 items using a 5-point Likert scale identical to that used in the original Sallis measure (1*=Never*, 2*=Rarely*, 3*=A few times*, 4*=Often*, 5*=Very often*, and 6*=Does not apply*, with scores of *6* being recoded as *1* before scoring). 

A total score for the SAM was created using the 10 support items and 10 accountability items. The Social Support subscale involves summing all social support items. Since the accountability items were measured on a 7-point scale and the social support items on a 5-point scale, the scores for the accountability measure involved summing all accountability items, multiplying the sum by 5, and then dividing by 7 to yield identical maximum scores for the 2 subscales. The total scores for social support and accountability were then summed to create an overall SAM score, with higher scores indicating higher levels of supportive accountability (possible range of total SAM scores 17.14-100). We then examined the psychometric characteristics of the SAM across 2 weight management trials (the study details are provided below under the Methods section for Study 1 and Study 2). Study procedures for Study 1 and Study 2 were approved by The Miriam Hospital Institutional Review Board, and secondary analyses of Study 2 data were approved by the University of Florida Institutional Review Board.

### Study 1

Study 1 evaluated the psychometric characteristics of the SAM in adults with obesity enrolled in a behavioral weight management trial. Only baseline data from this trial were used, allowing for the evaluation of the SAM in the relevant population before any intervention or support.

#### Participants

Study 1 included 353 adults (aged 40-60 years) with obesity (BMI between 30 kg/m^2^ and 40 kg/m^2^). Full eligibility criteria and recruitment procedures have been described elsewhere [20]. In brief, participants were recruited through mass mailings, newspaper/listserv ads, and direct referrals to an obesity treatment research center and were screened over the phone. Exclusion criteria were participation in another weight loss program, use of weight loss medication, history of bariatric surgery, weight loss of ≥5% in the last 6 months, pregnancy, lactation, less than 6 months postpartum, or plans to become pregnant during the study period, any health conditions that would contraindicate weight loss (eg, cancer, reported uncontrolled heart condition, inability to walk 2 blocks without stopping, and unexplained loss of consciousness), or inability to participate in an in-person, group program (eg, scheduling constraints). For individuals reporting joint problems, medication use, or other medical conditions that may limit the ability to exercise or need adjustment with weight loss, physicians’ consent was obtained. Study 1 participants were excluded from the current analyses if they did not complete the SAM measure at baseline (n=3).

#### Measures

All measures were collected at baseline.

##### Demographics

Standard demographics (eg, sex, age, race/ethnicity) were collected via a survey.

##### Anthropometrics

Height and weight were objectively assessed, with participants wearing one layer of light indoor clothing and shoes removed.

##### Supportive Accountability

The SAM was used to assess supportive accountability (see the Supportive Accountability Measure Development section for details).

##### Social Motivation for Weight Loss

The Treatment Self-Regulation Questionnaire (TSRQ) was administered to assess its convergent validity with the SAM. Given that the SAM is expected to measure social accountability, we examined whether the social external regulation subscale of the TSRQ [21] was associated with the SAM; 6 items were included, ranging from 1*=Not true at all* to 7*=Very true* (eg, “I want to lose weight or control my weight because…my spouse, family, friends or doctor would be upset if I didn’t”). Mean scores were calculated for this scale.

##### Social Pressure for Weight Loss

Social pressure was assessed using an item from the Motivating Factors for Weight Loss Scale, developed to assess motivation for weight loss among participants in the National Weight Control Registry [22]. Participants were asked how important social pressure was in their decision to lose weight or join a weight loss program. Response options ranged from 1*=Not at all important* to 5*=Extremely important*.

#### Statistical Analyses

Analyses were conducted using SPSS Statistics for Windows, version 25 (IBM Corp). The reliability of SAM items was assessed via the Cronbach alpha [23], and construct validity was assessed using an EFA using principal component analysis with Oblimin rotation (delta=0). A scree plot was used to assess the number of factors using the standard eigenvalue of *1* or greater. Convergent validity of the SAM was assessed by correlating total SAM scores with the total Social Motivation score and the Social Pressure item.

### Study 2

Study 2 was a randomized weight management trial investigating the impact of newer self-monitoring technology (ie, a Bluetooth-enabled activity monitor, a smart scale, and a website/smartphone app that synced with both of these devices and allowed individuals to self-monitor caloric intake) and phone coaching on weight loss. It was hypothesized that participants who received interventionist support through phone coaching would report significantly higher supportive accountability, as assessed by the SAM, at a 6-month posttest because of the presence of additional support. Moreover, we hypothesized that higher supportive accountability at intervention posttest would be associated with greater intervention adherence. As an exploratory aim, we investigated whether higher supportive accountability at the posttest was associated with greater weight loss from baseline to posttest.

#### Participants

Study 2 participants were 80 adults (aged between 18 and 70 years) with overweight or obesity (BMI between 27 kg/m^2^ and 40 kg/m^2^) who reported having access to a computer and Wi-Fi at home [24]. Exclusion criteria were similar to Study 1; full eligibility criteria and details regarding participant recruitment and screening have been published previously [24]. Study 2 participants were excluded from the current analyses if they did not complete the SAM measure at the 6-month follow-up.

#### Intervention

Study 2 was a randomized trial that examined the impact of a 6-month weight loss intervention in which participants were randomized to 1 of 3 treatment groups, using traditional self-monitoring tools (a paper food record, a printed calorie reference book, a standard pedometer, and a standard in-home scale—SC group), newer, technology-based self-monitoring tools (Fitbit Zip, Fitbit Aria smart scale, and use of the Fitbit app/website to track dietary intake—TECH group), or these newer technology tools plus phone-based interventionist support (TECH+PHONE group). All participants received a one-time, group-based Weight Loss 101 session that provided information on how to accurately monitor calories, weight, and physical activity and weight management goals for calories, exercise, and weight loss. Participants were also taught how to use their assigned self-monitoring tools. Participants randomized to SC (n=26) and TECH (n=27) received self-monitoring tools only; they did not receive any interventionist support. Participants randomized to TECH+PHONE (n=27) received the additional phone-based intervention (8 weekly, 4 biweekly, and 2 monthly contacts; each lasted 10-15 min), delivered by trained interventionists (either a clinical psychologist or dietitian, both experienced in delivering behavioral weight management programs), using a manualized protocol.

#### Measures

##### Demographics

Standard demographics (eg, sex, age, race/ethnicity) were collected via a survey at baseline.

##### Anthropometrics

Height and weight were measured with participants wearing light indoor clothing and with shoes removed. Height was measured at baseline and weight was measured at baseline and at the 6-month posttest. Weight change was operationalized as percent weight loss from baseline to the posttest visit.

##### Supportive Accountability

Supportive accountability was assessed at the 6-month posttest using the SAM.

##### Use of Weight Management Strategies

Weight management strategies were assessed at the 6-month posttest using the Weight Control Strategies Scale (WCSS) [25]. This self-report measure assesses behaviors across 4 subscales: dietary choices, self-monitoring strategies, physical activity, and psychological coping. WCSS dietary choices and physical activity subscales have been shown to be associated with changes in caloric intake and physical activity during weight management interventions, and WCSS scores have been shown to correlate with weight loss during these programs.

#### Statistical Analyses

Analyses were conducted using SPSS. Reliability was reassessed in this sample using Cronbach alpha. Construct validity of the SAM was assessed using a one-way analysis of variance, investigating differences in SAM scores by treatment group. We hypothesized that, at the 6-month posttest, the SAM score would be significantly higher in the TECH+PHONE condition than in the SC and TECH conditions, given that this condition was provided with additional interventionist support.

## Results

### Study 1

Study 1 included a total of 350 participants (Table 1). The mean SAM score was 48.63 (SD 16.33); mean support and accountability subscale scores were 22.71 (SD 7.80) and 36.28 (SD 17.28), respectively. There was an association between total SAM scores and baseline BMI (*r*=0.13; *P*=.02). In examining the subscales, accountability scores were positively associated with baseline BMI (*r*=0.17; *P*=.002), whereas support scores were not (*P*=.96). There were no associations between SAM scores and participant demographic characteristics (*P*>.06).

The overall reliability of the SAM was excellent, as demonstrated by the internal consistency (Cronbach α=.92). The lowest item to total SAM correlation (*r*=0.41) was for the item, “My friends/family encouraged me to not eat unhealthy foods when I’m tempted to do so.” However, the total Cronbach alpha was not improved with this item removed; thus, the item was retained. Cronbach alpha for the support and accountability subscales were also strong (.88 and .95, respectively). Inter-item correlations for the SAM are provided in Multimedia Appendix 2. As expected, items reflecting social support (items 1-10) correlated more closely with each other and had lower correlations with accountability items (items 11-20).

**Figure 1:**
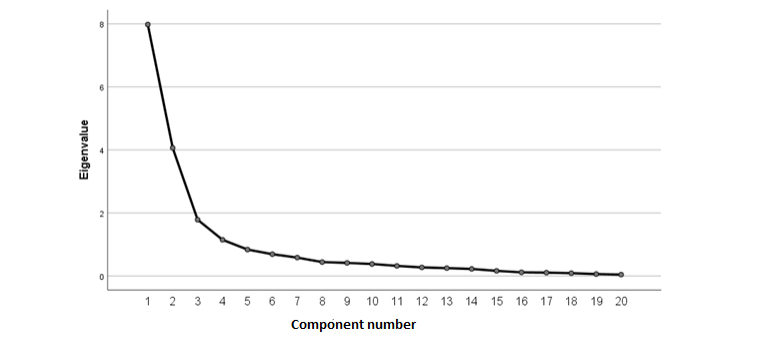
Scree plot for exploratory factor analysis of the supportive accountability measure.

Results from the EFA demonstrated that a three-factor solution provided the best fit (Figure 1), explaining 69.2% of the variance. Item loadings for the three-factor solution, representing Support for Healthy Eating Habits, Support for Exercise Habits, and Perceptions of Accountability, are presented in Table 2. Assumptions of EFA were verified using the Kaiser-Meyer-Olkin (KMO) statistic, which demonstrated sampling adequacy (KMO=0.88). Furthermore, the Bartlett test of sphericity demonstrated that items correlated satisfactorily (*χ^2^*_190_=6573.91; *P*<.001).

Convergent and divergent validity analyses revealed statistically significant correlations between the SAM total score and TSRQ items representing external motivation for weight loss (*r*=0.34; *P*<.001) and the social pressure item (*r*=0.23; *P*<.001). In examining the subscales of SAM, there were significant correlations between the Support subscale and Social Motivation items (*r=*0.19; *P*=.001) and the Social Pressure item (*r*=0.17; *P*=.002). Similarly, there were significant correlations between the Perceptions of Accountability subscale and Social Motivation items (*r*=0.33; *P*<.001) and the Social Pressure item (*r*=0.20; *P*<.001).

**Table 1:** Baseline and demographic characteristics of participants in study 1

Characteristic		Study 1 (n=350)
Age (years), mean (SD)		51.7 (5.6)
BMI (kg/m^2^), mean (SD)		34.8 (3.3)
**Gender, n (%)**		
	Male	80 (22.9)
	Female	270 (77.1)
**Education, n (%)**		
	High school or less	38 (10.9)
	Vocational training	24 (6.9)
	Some college	83 (23.7)
	College degree	108 (30.9)
	Graduate degree	97 (27.7)
**Race, n (%)**		
	American Indian or Alaska Native	6 (1.7)
	Asian	1 (0.3)
	Black or African American	36 (10.3)
	White	262 (74.9)
	Other	36 (10.3)
**Ethnicity, n (%)**		
	Hispanic or Latino	45 (12.9)
	Not Hispanic or Latino	304 (86.9)

**Table 2:** Three-factor structure for Supportive Accountability Measure items.

Items		Support for Healthy Eating	Support for Exercise Habits	Perceptions of Accountability
**My weight coach, friends, and/or family**				
	1. Encouraged me not to eat *unhealthy foods* (cake, potato chips) when I’m tempted to do so	0.81	N/A^a^	N/A
	2. Discussed my eating habit changes with me (asked me how I’m doing with my eating changes)	0.83	N/A	N/A
	3. Reminded me not to eat high fat, high calorie foods	0.85	N/A	N/A
	4. Complimented me on changing my eating habits (“Keep it up. We are proud of you.”)	0.68	N/A	N/A
	5. Commented if I went back to my old eating habits	0.71	N/A	N/A
	6. Exercised with me	N/A	0.88	N/A
	7. Offered to exercise with me	N/A	0.87	N/A
	8. Gave me helpful reminders to exercise (“Are you going to exercise tonight?”)	N/A	0.85	N/A
	9. Gave me encouragement to stick with my exercise program	N/A	0.78	N/A
	10. Changed their schedule so we could exercise together	N/A	0.81	N/A
	11. I feel accountable to others (eg, friends, family, or doctor) for meeting my weight goals.	N/A	N/A	0.77
	12. I feel accountable to others (eg, friends, family, or doctor) for meeting my dietary goals.	N/A	N/A	0.79
	13. I feel accountable to others (eg, friends, family, or doctor) for meeting my exercise goals.	N/A	N/A	0.79
	14. I feel that I would let others down (eg, friends, family, or doctor) if I did not meet my weight goals.	N/A	N/A	0.93
	15. I feel that I would let others down (eg, friends, family, or doctor) if I did not meet my dietary goals.	N/A	N/A	0.94
	16. I feel that I would let others down (eg, friends, family, or doctor) if I did not meet my exercise goals.	N/A	N/A	0.92
	17. Feeling accountable to others (eg, friends, family, or doctor) has helped me control my weight.	N/A	N/A	0.57
	18. Feeling accountable to others (eg, friends, family, or doctor) has helped me stay on track with my diet.	N/A	N/A	0.55
	19. Feeling accountable to others (eg, friends, family, or doctor) has helped me stay on track with my physical activity.	N/A	N/A	0.54
	20. In general, I feel accountable to others to control my weight.	N/A	N/A	0.74

^a^N/A: not applicable.

### Study 2

Of the 80 participants included in Study 2, 55 completed the SAM at the 6-month assessment and were thus included in these analyses. Table 3 provides participants’ baseline characteristics by group. There were no significant differences between groups in terms of age, baseline BMI, sex, education, or race/ethnicity, all *P* values were >.05.

At the 6-month follow-up, the mean SAM score was 47.86 (SD 20.88); scores ranged from 17.14 to 94.57 (highest possible score of 100). Replicating the analyses conducted in Study 1, the reliability analyses for 6-month SAM scores demonstrated strong internal consistency (Cronbach α=.95).

**Table 3:** Baseline and demographic characteristics of participants by group in study 2.

Characteristic		Study 2 (n=55)		
		SC^a^ (n=17)	TECH^b^ (n=17)	TECH+PHONE^c^ (n=21)
Age (years), mean (SD)		54.9 (9.7)	46.4 (12.7)	52.9 (11.28)
BMI (kg/m^2^), mean (SD)		34.0 (4.0)	33.0 (3.4)	32.2 (3.3)
**Gender, n (%)**				
	Male	4 (23)	2 (11)	4 (19)
	Female	13 (76)	15 (88)	17 (81)
**Education, n (%)**				
	High school or less	1 (5)	0 (0)	2 (9)
	Vocational training	0 (0)	1 (5)	0 (0)
	Some college	4 (23)	3 (17)	5 (23)
	College degree	6 (35)	7 (41)	6 (28)
	Graduate degree	6 (35)	6 (35)	8 (38)
**Race, n (%)**				
	American Indian or Alaska Native	0 (0)	0 (0)	0 (0)
	Asian	0 (0)	0 (0)	0 (0)
	Black or African American	2 (11)	2 (11)	0 (0)
	White	15 (88)	15 (88)	21 (100)
	Other	0 (0)	0 (0)	0 (0)
**Ethnicity, n (%)**				
	Hispanic or Latino	0 (0)	1 (6)	1 (4)
	Not Hispanic or Latino	17 (100)	16 (94)	20 (95)

^a^SC: standard in-home scale group.

^b^TECH: technology-based self-monitoring tools group.

^c^TECH+PHONE: technology-based self-monitoring tools plus phone-based support group.

As hypothesized, there were significant differences in the 6-month total SAM scores by treatment group (*F*_2,52_=20.9; *r^2^*=0.45; *P*<.001; Table 4). Bonferroni-adjusted posthoc analyses demonstrated significantly higher SAM scores in the TECH+PHONE group than the TECH group (t_54_=3.70, *P*<.001; SC: t_54_=6.39, *P*<.001). Moreover, the TECH group demonstrated significantly higher SAM scores compared with SC (t_54_=2.57; *P*=.04).

Between-group differences in SAM subscales were also examined (Table 4). The Support for Healthy Eating subscale demonstrated a similar pattern to the overall SAM scores. There was a significant difference in Support for Healthy Eating subscale scores by group (*F*_2,52_=21.4; *r^2^*=0.45; *P*<.001), with the TECH+PHONE group demonstrating higher subscale scores than both TECH (t_54_=3.48; *P*<.001) and SC (t_54_=6.51; *P*<.001) groups, and the TECH group demonstrating significantly higher scores compared with the SC group (t_54_=2.88; *P*=.02). Although an overall significant difference between groups was demonstrated on the Support for Exercise Habits subscale (*F*_2,52_=3.9; *r^2^*=0.13; *P*=.03), there were no significant group differences when conducting Bonferroni-corrected posthoc comparisons. Finally, there was a significant difference between groups in the Perceptions of Accountability subscale (*F*_2,52_=15.3; *r^2^*=0.37; *P*<.001), such that the TECH+PHONE group had higher scores on this subscale compared with both TECH (t_54_=3.96; *P*<.001) and SC (t_54_=5.22; *P*<.001) groups. There were no significant differences in Perceptions of Accountability subscale scores between TECH and SC (*P*=.71) groups.

Table 5 presents correlations between SAM scores and adherence to weight management behaviors and percent weight change during the intervention. Higher SAM scores were associated with higher adherence to weight management behaviors, including higher scores on subscales representing healthy dietary choices, the use of self-monitoring strategies, and positive psychological coping with weight management challenges. The association between total SAM scores and percent weight change was in the expected direction but not statistically significant (*r*=−0.26; *P*=.06).

**Table 4:** Six-month scores on supportive accountability measure (SAM) and each of the three SAM subscales by intervention group.

Scale		Value, mean (SE)	
**Supportive accountability measure**			
	SC^a^		30.9 (3.8)
	TECH^b^		44.9 (3.8)
	TECH+PHONE^c^		64.0 (3.5)
**Factor 1: Support for Healthy Eating**			
	SC		9.8 (1.2)
	TECH		14.5 (1.2)
	TECH+PHONE		20.0 (1.1)
**Factor 2: Support for Exercise**			
	SC		9.2 (1.4)
	TECH		14.0 (1.4)
	TECH+PHONE		13.7 (1.2)
**Factor 3: Perceptions of Accountability**			
	SC		11.9 (2.6)
	TECH		16.3 (2.6)
	TECH+PHONE		30.3 (2.3)

^a^SC: standard in-home scale group.

^b^TECH: technology-based self-monitoring tools group.

^c^TECH+PHONE: technology-based self-monitoring tools plus phone-based support group.

**Table 5:** Correlation between the supportive accountability measure (total scores and subscales) and adherence to weight control strategies and weight change from baseline to 6-month posttest.

Scale		Total SAM^a^ score				Support for Healthy Eating Habits			Support for Exercise				Perceptions of Accountability		
		*r*		*P* value		*r*		*P* value	*r*		*P* value		*r*	*P* value	
**Weight Control Strategies Scale total score**		0.47		.003^b^		0.26		.06	0.23		.09		0.50	<.001^b^	
	Dietary choices		0.29		.03^b^		0.14	.30		0.15		.27	0.32		.01^b^
	Self-monitoring strategies		0.47		.003^b^		0.28	.03^b^		0.26		.06	0.49		.002^b^
	Physical activity		0.23		.08		−0.02	.90		0.15		.28	0.31		.02^b^
	Psychological coping		0.45		.007^b^		0.36	.006^b^		0.16		.25	0.46		<.001^b^
Percent weight change from baseline to 6 months		−0.26		.06		−0.21		.11	−0.12		.39		−0.25	.06	

^a^SAM: supportive accountability measure.

^b^Statistically significant (*P*<.05).

## Discussion

### Study 1

Study 1 evaluated the psychometric properties of the novel, theory-based SAM in a sample of adults interested in a behavioral weight management trial. EFA revealed a three-factor solution for the SAM, representing subscales for Support for Healthy Eating Habits, Support for Exercise Habits, and Perceptions of Accountability. All items were retained, leaving 10 items representing social support and 10 items representing accountability, which together form the theoretical basis for the construct of supportive accountability [17]. Overall, the 20-item SAM demonstrated excellent internal consistency. Moreover, SAM total scores were significantly associated with measures of Social Motivation [21] and Social Pressure [22]. However, these correlations were small to moderate in magnitude, consistent with the idea that supportive accountability is a construct distinct from motivation related to fears of upsetting others or feelings of social pressure [17]. Examining the convergent validity of the SAM with other measures of social support with weight loss may yield higher correlations. Overall, SAM showed strong reliability and validity in adults with obesity seeking weight loss.

### Study 2

Study 2 evaluated the criterion validity of the SAM. Consistent with the hypotheses, participants provided with additional phone support (TECH+PHONE) reported higher SAM scores at the end of a 6-month weight loss program compared with participants who did not receive phone support (TECH only or SC only). Interestingly, participants who did not receive phone support but were provided with newer, technology-based self-monitoring tools reported higher supportive accountability at the end of the intervention compared with participants randomized to self-monitoring using traditional tools (a standard pedometer, bathroom scale, a calorie reference book, and paper self-monitoring logs used to track physical activity, weight, and caloric intake). Subscale analyses revealed that this was likely driven by increased feelings of social support but not accountability. It may be that the brief, automated feedback provided by these tools gave participants a sense of support. Specifically, these tools provided immediate feedback to participants who met short- and long-term goals (such as notifications on the activity monitor and pushed smartphone notifications and *badges* displayed on the app homepage that functioned as visual reminders of goals that had been met) [24]. Similarly, tailored feedback related to intervention goals, even when provided by automated systems, has been demonstrated to improve weight loss outcomes [10]. Thus, there may be some *digital support* inherent in this feedback. Additionally, the availability of social/community features on the Fitbit platforms (eg, the ability to share step counts with friends, competitions among friends to achieve certain step goals) might have promoted feelings of social support. The parent study for Study 2 was conducted between and 2013 and 2015 when there were only minimal social components to these tools. Future research should investigate whether newer, more comprehensive social/community features further lead to increased feelings of support.

Consistent with the Supportive Accountability Theory [10], the provision of additional phone-based support resulted in the highest SAM scores. Compared with the 2 groups that did not receive phone support (TECH and SC), participants provided with phone support demonstrated significantly higher SAM total scores and subscale scores for Support for Healthy Eating Habits and Perceptions of Accountability. This suggests that ongoing interventionist contact improves support for healthy eating and provides a sense of accountability toward weight management behaviors and weight loss goals. Although the overall test statistic demonstrated significant differences across groups in Support for Exercise Habits, there were no posthoc group differences between any of the groups after Bonferroni corrections. This may have been related to power (Bonferroni corrections represent the most conservative approach but can lead to type II errors, especially in small samples [25]). Moreover, it may be possible that the items on this subscale were influenced less by the type of intervention utilized in Study 2. It would not be expected that items asking about whether supportive individuals “exercised with me,” “offered to exercise with me,” and “changed their schedule so we could exercise together” would be rated higher in participants receiving additional phone-based interventionist support. Thus, future research may investigate whether alternative item wording for exercise may better reflect the social support provided by intervention staff.

Finally, consistent with Mohr’s theory that supportive accountability could increase intervention adherence [16], the results demonstrated that higher SAM scores were associated with better adherence to weight management strategies. Interestingly, these associations appear to be driven by ratings of accountability versus support. The correlations between the Perceptions of Accountability subscale and report of adherence to weight management behaviors on the WCSS (both the WCSS total score and all subscale scores) were larger than associations between weight management adherence and the social support subscales (either Support for Healthy Eating Habits or Support for Exercise Habits). Although there was no statistically significant association between total SAM score and percent weight change during the intervention, the association was in the expected direction and represented a medium effect size. Similarly, the association between the SAM subscales (Support for Healthy Eating, Support for Exercise, and Perceptions of Accountability) and percent weight change was not statistically significant; however, it was in the expected direction.

Overall, data from 2 studies were used to examine the psychometric properties of the SAM, a new survey to assess supportive accountability for weight management behaviors. Across both studies, the SAM demonstrated excellent internal consistency and construct validity. This study has some limitations. Participants were predominantly female and non-Hispanic white, which limits the generalizability of the study results. Future research is needed to examine whether SAM demonstrates a similar factor structure, internal consistency, and validity in more diverse samples. The small sample size in Study 2, combined with the fact that participants in only 2 of the 3 groups were asked to use technology-based self-monitoring tools, precluded the investigation into whether the SAM was associated with objective engagement with the technology-based self-monitoring tools. This study also did not assess test-retest reliability or sensitivity to change over time. Future work in these areas would strengthen confidence in this measure for assessing the construct of supportive accountability and further provide important results that could inform future theory development. Finally, the scoring on this measure was complicated by the different scales used for scoring the accountability items (which used a 7-point scale) and the social support items (which used a 5-point scale). The accountability items were developed before the selection of social support items from the Sallis questionnaire, and a 7-point scale was chosen to optimize variability in responses. Future research should investigate whether this scale performs similarly when all items use the same scale (eg, either 5 or 7 response points).

This research also has notable strengths. The development of SAM was theory-based, relying on the Supportive Accountability Theory by Mohr [17]. The SAM included the use of a widely used validated measure of social support as a basis [18] and built upon this measure by adding psychometrically sound accountability items. Furthermore, reducing the items from the original social support scale makes this new tool a concise measure of support and accountability that can be used in many technology-mediated interventions that would avoid scale fatigue and respondent burden. In Study 1, the sample size was large, which allowed for initial validation and an EFA. Furthermore, all weight data were objectively assessed. Moreover, in 2 separate samples, the SAM demonstrated both internal consistency and construct validity. Finally, the SAM was included in a randomized trial that involved interventions with and without phone support, which allowed for the examination of criterion validity. These methodologies allowed for a robust examination of the psychometric properties of a new measure of supportive accountability.

Supportive accountability was developed as a construct within the context of technology-mediated *electronic health* interventions [16]; however, this construct may have broader applicability. For example, it is theoretically plausible that supportive accountability may play a role in promoting adherence even in traditional face-to-face behavioral interventions. With the SAM, future studies will be able to investigate whether supportive accountability mediates outcomes in face-to-face programs. This may be particularly important in interventions that rely heavily on social support.

Moreover, although the construct of supportive accountability suggests the importance of human support [16], the SAM would be useful for research focused on the development of technologically mediated support systems. For example, the SAM could be used to evaluate whether tools that integrate social components (eg, leaderboards, competitions among friend groups, or chat rooms/bulletin boards that allow contacts to provide support) increase feelings of supportive accountability and ultimately promote intervention adherence. The SAM could also be used to evaluate whether automated feedback provided via tailoring algorithms or artificial intelligence programs can impact feelings of supportive accountability.

Considering the ever-growing technological innovations, the SAM will help researchers better understand the factors that drive the effectiveness of technology-based treatments. This use of the SAM may thus guide the development of more effective interventions and help improve foundational knowledge regarding the mechanisms that drive treatment effects in technologically mediated treatment.

## Appendix

Multimedia Appendix 1 The supportive accountability measure and scoring.

Multimedia Appendix 2 Inter-item correlations between supportive accountability measure items.

Multimedia Appendix 3 CONSORT-eHEALTH checklist (V 1.6.2).

## Abbreviations

EFA: exploratory factor analysis
KMO: Kaiser-Meyer-Olkin
SAM: Supportive Accountability Measure
TSRQ: Treatment Self-Regulation Questionnaire
WCSS: Weight Control Strategies Scale

## References

[1] Raaijmakers LC, Pouwels S, Berghuis KA, Nienhuijs SW (2015). Technology-based interventions in the treatment of overweight and obesity: a systematic review. Appetite.

[2] Lin H, Wu X (2014). Intervention strategies for improving patient adherence to follow-up in the era of mobile information technology: a systematic review and meta-analysis. PLoS One.

[3] Gell NM, Grover KW, Humble M, Sexton M, Dittus K (2017). Efficacy, feasibility, and acceptability of a novel technology-based intervention to support physical activity in cancer survivors. Support Care Cancer.

[4] Plotnikoff RC, Wilczynska M, Cohen KE, Smith JJ, Lubans DR (2017). Integrating smartphone technology, social support and the outdoor physical environment to improve fitness among adults at risk of, or diagnosed with, Type 2 Diabetes: findings from the 'eCoFit' randomized controlled trial. Prev Med.

[5] Shuger SL, Barry VW, Sui X, McClain A, Hand GA, Wilcox S, Meriwether RA, Hardin JW, Blair SN (2011). Electronic feedback in a diet- and physical activity-based lifestyle intervention for weight loss: a randomized controlled trial. Int J Behav Nutr Phys Act.

[6] Lynch SM, Stricker CT, Brown JC, Berardi JM, Vaughn D, Domchek S, Filseth S, Branas A, Weiss-Trainor E, Schmitz KH, Sarwer DB (2017). Evaluation of a web-based weight loss intervention in overweight cancer survivors aged 50 years and younger. Obes Sci Pract.

[7] Coons MJ, Demott A, Buscemi J, Duncan JM, Pellegrini CA, Steglitz J, Pictor A, Spring B (2012). Technology interventions to curb obesity: a systematic review of the current literature. Curr Cardiovasc Risk Rep.

[8] Leahey TM, Thomas G, Fava JL, Subak LL, Schembri M, Krupel K, Kumar R, Weinberg B, Wing RR (2014). Adding evidence-based behavioral weight loss strategies to a statewide wellness campaign: a randomized clinical trial. Am J Public Health.

[9] Norman GJ, Zabinski MF, Adams MA, Rosenberg DE, Yaroch AL, Atienza AA (2007). A review of eHealth interventions for physical activity and dietary behavior change. Am J Prev Med.

[10] Kozak AT, Buscemi J, Hawkins MA, Wang ML, Breland JY, Ross KM, Kommu A (2017). Technology-based interventions for weight management: current randomized controlled trial evidence and future directions. J Behav Med.

[11] Jensen MD, Ryan DH, Apovian CM, Ard JD, Comuzzie AG, Donato KA, Hu FB, Hubbard VS, Jakicic JM, Kushner RF, Loria CM, Millen BE, Nonas CA, Pi-Sunyer FX, Stevens J, Stevens VJ, Wadden TA, Wolfe BM, Yanovski SZ, American College of Cardiology/American Heart Association Task Force on Practice Guidelines, Obesity Society (2014). 2013 AHA/ACC/TOS guideline for the management of overweight and obesity in adults: a report of the American College of Cardiology/American Heart Association Task Force on Practice Guidelines and The Obesity Society. J Am Coll Cardiol.

[12] Ryan K, Dockray S, Linehan C (2019). A systematic review of tailored eHealth interventions for weight loss. Digit Health.

[13] Butryn ML, Webb V, Wadden TA (2011). Behavioral treatment of obesity. Psychiatr Clin North Am.

[14] Andersson G, Cuijpers P (2009). Internet-based and other computerized psychological treatments for adult depression: a meta-analysis. Cogn Behav Ther.

[15] Lemstra M, Bird Y, Nwankwo C, Rogers M, Moraros J (2016). Weight loss intervention adherence and factors promoting adherence: a meta-analysis. Patient Prefer Adherence.

[16] Tate DF, Jackvony EH, Wing RR (2003). Effects of Internet behavioral counseling on weight loss in adults at risk for type 2 diabetes: a randomized trial. J Am Med Assoc.

[17] Mohr DC, Cuijpers P, Lehman K (2011). Supportive accountability: a model for providing human support to enhance adherence to eHealth interventions. J Med Internet Res.

[18] Sallis JF, Grossman RM, Pinski RB, Patterson TL, Nader PR (1987). The development of scales to measure social support for diet and exercise behaviors. Prev Med.

[19] Kiernan M, Moore SD, Schoffman DE, Lee K, King AC, Taylor CB, Kiernan NE, Perri MG (2012). Social support for healthy behaviors: scale psychometrics and prediction of weight loss among women in a behavioral program. Obesity (Silver Spring).

[20] Leahey TM, Huedo-Medina T, Grenga A, Gay L, Fernandes Pierre D, Denmat Z, Areny R, Wing RR Patient provided e-support for reduced intensity obesity treatment: The INSPIRE Trial. Health Psychology..

[21] Levesque CS, Williams GC, Elliot D, Pickering MA, Bodenhamer B, Finley PJ (2007). Validating the theoretical structure of the Treatment Self-Regulation Questionnaire (TSRQ) across three different health behaviors. Health Educ Res.

[22] LaRose JG, Leahey TM, Hill JO, Wing RR (2013). Differences in motivations and weight loss behaviors in young adults and older adults in the National Weight Control Registry. Obesity (Silver Spring).

[23] Boateng GO, Neilands TB, Frongillo EA, Melgar-Quiñonez HR, Young SL (2018). Best practices for developing and validating scales for health, social, and behavioral research: a primer. Front Public Health.

[24] Ross KM, Qiu P, You L, Wing RR (2018). Characterizing the pattern of weight loss and regain in adults enrolled in a 12-week internet-based weight management program. Obesity (Silver Spring).

[25] Pinto AM, Fava JL, Raynor HA, LaRose JG, Wing RR (2013). Development and validation of the weight control strategies scale. Obesity (Silver Spring).

[26] Nakagawa S (2004). A farewell to Bonferroni: the problems of low statistical power and publication bias. Behav Ecol.

