# Supplementary material for: The Assessment of Supportive Accountability in Adults Seeking Obesity Treatment: Psychometric Validation Study
Source: J Med Internet Res. 2020 Jul 28;22(7):e17967. doi: 10.2196/17967 (PMC7420735; doi:10.2196/17967)
Supplement: Multimedia Appendix 1 [file jmir_v22i7e17967_app1.DOC]

**Supportive Accountability Measure**

Directions: Below is a list of things people might do or say to someone who is trying to improve their eating and activity habits. Please rate how often people around you (weight coach, friends, family, etc.) have said or done what is described.

| My weight coach, friends, and/or family…. | Never | Rarely | A few times | Often | Very often | Does not apply |
| --- | --- | --- | --- | --- | --- | --- |
| 1. Encouraged me not to eat “unhealthy foods” (cake, potato chips) when I’m tempted to do so | 1 | 2 | 3 | 4 | 5 | 6 |
| 2. Discussed my eating habit changes with me (asked me how I’m doing with my eating changes) | 1 | 2 | 3 | 4 | 5 | 6 |
| 3. Reminded me not to eat high fat, high calorie foods | 1 | 2 | 3 | 4 | 5 | 6 |
| 4. Complimented me on changing my eating habits (“Keep it up. We are proud of you.”) | 1 | 2 | 3 | 4 | 5 | 6 |
| 5. Commented if I went back to my old eating habits | 1 | 2 | 3 | 4 | 5 | 6 |
| 6. Exercised with me | 1 | 2 | 3 | 4 | 5 | 6 |
| 7. Offered to exercise with me | 1 | 2 | 3 | 4 | 5 | 6 |
| 8. Gave me helpful reminders to exercise (“Are you going to exercise tonight?”) | 1 | 2 | 3 | 4 | 5 | 6 |
| 9. Gave me encouragement to stick with my exercise program | 1 | 2 | 3 | 4 | 5 | 6 |
| 10. Changed their schedule so we could exercise together | 1 | 2 | 3 | 4 | 5 | 6 |

| 1. I feel accountable to others (e.g., coach, friends, family, doctor) for meeting my weight goals. | | | | | | |
| --- | --- | --- | --- | --- | --- | --- |
| ➀ | ➁ | ➂ | ➃ | ➄ | ➅ | ➆ |
| Not at all |  |  |  |  |  | Very much |
| 1. I feel accountable to others (e.g., coach, friends, family, doctor) for meeting my dietary goals. | | | | | | |
| ➀ | ➁ | ➂ | ➃ | ➄ | ➅ | ➆ |
| Not at all |  |  |  |  |  | Very much |
| 1. I feel accountable to others (e.g., coach, friends, family, doctor) for meeting my exercise goals. | | | | | | |
| ➀ | ➁ | ➂ | ➃ | ➄ | ➅ | ➆ |
| Not at all |  |  |  |  |  | Very much |
| 1. I feel that I would let others down (e.g., coach, friends, family, doctor) if I did not meet my weight goals. | | | | | | |
| ➀ | ➁ | ➂ | ➃ | ➄ | ➅ | ➆ |
| Not at all |  |  |  |  |  | Very much |
| 1. I feel that I would let others down (e.g., coach, friends, family, doctor) if I did not meet my dietary goals. | | | | | | |
| ➀ | ➁ | ➂ | ➃ | ➄ | ➅ | ➆ |
| Not at all |  |  |  |  |  | Very much |
| 1. I feel that I would let others down (e.g., coach, friends, family, doctor) if I did not meet my exercise goals. | | | | | | |
| ➀ | ➁ | ➂ | ➃ | ➄ | ➅ | ➆ |
| Not at all |  |  |  |  |  | Very much |
| 1. Feeling accountable to others (e.g., coach, friends, family, doctor) has helped me control my weight. | | | | | | |
| ➀ | ➁ | ➂ | ➃ | ➄ | ➅ | ➆ |
| Not at all |  |  |  |  |  | Very much |
| 1. Feeling accountable to others (e.g., coach, friends, family, doctor) has helped me stay on track with my diet. | | | | | | |
| ➀ | ➁ | ➂ | ➃ | ➄ | ➅ | ➆ |
| Not at all |  |  |  |  |  | Very much |
| 1. Feeling accountable to others (e.g., coach, friends, family, doctor) has helped me stay on track with my physical activity. | | | | | | |
| ➀ | ➁ | ➂ | ➃ | ➄ | ➅ | ➆ |
| Not at all |  |  |  |  |  | Very much |
| 1. In general, I feel accountable to others to control my weight. | | | | | | |
| ➀ | ➁ | ➂ | ➃ | ➄ | ➅ | ➆ |
| Not at all |  |  |  |  |  | Very much |

Scoring: The SAM consists of two subscales and a total score. The Social Support subscale involves summing all social support items (items 1-10). The Accountability subscale involves summing all accountability items, multiplying the sum by 5, and then dividing by 7 (items 11-20); such an approach yields identical maximum scoring for the two subscales, due to difference in Likert scales. The total scores for Social Support and Accountability are then summed to create an overall SAM score, with higher scores indicating higher levels of Supportive Accountability (possible range of total SAM scores 17.14 – 100).
