# Supplementary figures and images for: The Assessment of Supportive Accountability in Adults Seeking Obesity Treatment: Psychometric Validation Study
Source: J Med Internet Res. 2020 Jul 28;22(7):e17967. doi: 10.2196/17967 (PMC7420735; doi:10.2196/17967)

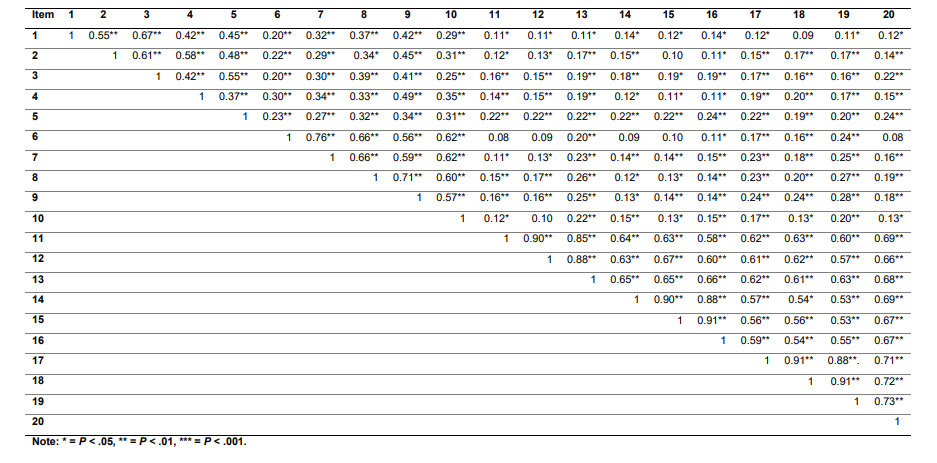

Supplement: Multimedia Appendix 2 [file jmir_v22i7e17967_app2.png]
