# Supplementary material for: The Assessment of Supportive Accountability in Adults Seeking Obesity Treatment: Psychometric Validation Study
Source: J Med Internet Res. 2020 Jul 28;22(7):e17967. doi: 10.2196/17967 (PMC7420735; doi:10.2196/17967)
Supplement: Multimedia Appendix 3 [file jmir_v22i7e17967_app3.pdf]

CONSORT-EHEALTH Checklist V1.6.2 Report	Manuscript Number	17967
**(based on CONSORT-EHEALTH V1.6), available at [http://tinyurl.com/consort-ehealth-v1-6].**		
**Date completed**		
6/29/2020 6:14:55		
**by**		
Margrethe Røed		
Process Evaluation of an eHealth Intervention (Food4toddlers) to Improve Toddler's Diet: Randomized Controlled Trial		
TITLE		
**1a-i) Identify the mode of delivery in the title**		
eHealth intervention		
**1a-ii) Non-web-based components or important co-interventions in title**		
Process Evaluation		
**1a-iii) Primary condition or target group in the title**		
NA		
ABSTRACT		
**1b-i) Key features/functionalities/components of the intervention and comparator in the METHODS section of the ABSTRACT**		
"process evaluation", "electronic health (eHealth) intervention", "website usage"		
**1b-ii) Level of human involvement in the METHODS section of the ABSTRACT**		
"parent–toddler dyads", "parents in the intervention group received access to an intervention website for 6 months"		
**1b-iii) Open vs. closed, web-based (self-assessment) vs. face-to-face assessments in the METHODS section of the ABSTRACT**		
Mentioned in the method section.		
**1b-iv) RESULTS section in abstract must contain use data**		
The total number of the trial and for this analysis was mentioned in the method section. The results of primary (satisfaction and usages of the website) and secondary (sub-group analyzes) is mentioned.		
**1b-v) CONCLUSIONS/DISCUSSION in abstract for negative trials**		
The overall results of the prosess evaluation is mentioned(was relevant for the participants) and the secondary outcomes (differences according to education level and family composition).		
INTRODUCTION		
**2a-i) Problem and the type of system/solution**		
"Internet is a powerful and popular source for health information among parents"<br>"Mobile health (mHealth) and eHealth interventions are gaining popularity, as such interventions have the potential to reach a large target group, can easily be adapted to new groups, are available 24/7, and can be cost-effective"		
**2a-ii) Scientific background, rationale: What is known about the (type of) system**		
We report results for prosess evaluations of similar interventions e.g. the Early Food for Future Health study and the Growing Healhty Program. In adition to describe the potential and interest of developing and evaluating the usage and satisfaction of this type of interventions.		
**Does your paper address CONSORT subitem 2b?**		
"The objectives of this study were to conduct a process evaluation of this eHealth intervention by examining the usage and perceived satisfaction of the<br>intervention website among parents of toddlers and to explore whether this differed according to educational level and number of children in the household."		
METHODS		
**3a) CONSORT: Description of trial design (such as parallel, factorial) including allocation ratio**		
"Food4toddlers is a randomized controlled trial, aiming to promote healthy dietary habits among toddlers"		
**3b) CONSORT: Important changes to methods after trial commencement (such as eligibility criteria), with reasons**		
NA		
**3b-i) Bug fixes, Downtimes, Content Changes**		
NA		
**4a) CONSORT: Eligibility criteria for participants**		
"Eligible individuals were parents of children born between June 2016 and May 2017. The parents had to be literate in Norwegian"		
**4a-i) Computer / Internet literacy**		
"Eligible individuals were parents of children born between June 2016 and May 2017. The parents had to be literate in Norwegian"<br>No other eligibility criteria was applied.		
**4a-ii) Open vs. closed, web-based vs. face-to-face assessments:**		
We have stated that the parents were recruited through Facebook, and signed in on a website. Online questionnaires were used for assessment.		
**4a-iii) Information giving during recruitment**		
"A total of 404 parents of 12-month-old children were recruited through a Facebook advertisement, who then responded to a baseline questionnaire and were randomized into an intervention group and a control group".		
**4b) CONSORT: Settings and locations where the data were collected**		
Norway (Facebook)		
**4b-i) Report if outcomes were (self-)assessed through online questionnaires**		
The mode of delivery of the intervetion was not stated in the section, however there was a link to the protocol paper listed.		
**4b-ii) Report how institutional affiliations are displayed**		
All procedures were reviewed and approved by the NSD (Norwegian centre for research data), and informed consent from the parents was obtained when they signed in online for participation in the study.		
**5) CONSORT: Describe the interventions for each group with sufficient details to allow replication, including how and when they were actually administered**		
**5-i) Mention names, credential, affiliations of the developers, sponsors, and owners**		
"The authors thank the participants. This study is funded by the University of Agder."		
**5-ii) Describe the history/development process**		
This paper is a process evaluation and such details are presented in the protocol paper of for the intervention (https://doi.org/10.1186/doi s12889-019-6915-x).		
**5-iii) Revisions and updating**		
NA		
**5-iv) Quality assurance methods**		
NA		
**5-v) Ensure replicability by publishing the source code, and/or providing screenshots/screen-capture video, and/or providing flowcharts of the algorithms used**		
"The intervention group had 6 months of access to the Food4toddlers website which comprised 4 main elements: (1) lessons (n=22) on how to provide healthy food and create a healthy eating environment for the toddler, (2) recipes, (3) a discussion forum, and (4) basic information about food and beverages (called Good to know)".		
**5-vi) Digital preservation**		
NA. (The content will be developed on another digital platform in the future)		
**5-vii) Access**		

"Participants in the intervention group were<br>given access to the Food4toddlers website for 6 months". More details are presented in the protocol paper of for the intervention<br>(https://doi.org/10.1186/doi s12889-019-6915-x).		
**5-viii) Mode of delivery, features/functionalities/components of the intervention and comparator, and the theoretical framework**		
These issues are presented in the protocol paper of for the intervention<br>(https://doi.org/10.1186/doi s12889-019-6915-x).		
**5-ix) Describe use parameters**		
The duration of the intervention is listed (6 months) in the Intervention development section along with the content.<br>5-		
**5-x) Clarify the level of human involvement**		
The duration of the intervention is listed (6 months) and no tailoring except for push notivifactions were awailible.		
**5-xi) Report any prompts/reminders used**		
"Initially, the web page was<br>limited to information relevant for the child's age at baseline<br>and gradually expanded in 20 steps as the child got older."		
**5-xii) Describe any co-interventions (incl. training/support)**		
NA		
**6a) CONSORT: Completely defined pre-specified primary and secondary outcome measures, including how and when they were assessed**		
Stated in the Data and Measurements and statistics section.		
**6a-i) Online questionnaires: describe if they were validated for online use and apply CHERRIES items to describe how the questionnaires were designed/deployed**		
All questions used are validated and reliability tested. Stated in the "Measures and<br>outcomes" section.		
**6a-ii) Describe whether and how "use" (including intensity of use/dosage) was defined/measured/monitored**		
THis is the main results of this article. Se the results section.		
**6a-iii) Describe whether, how, and when qualitative feedback from participants was obtained**		
NA		
**6b) CONSORT: Any changes to trial outcomes after the trial commenced, with reasons**		
Norway (Facebook)		
**7a) CONSORT: How sample size was determined**		
**7a-i) Describe whether and how expected attrition was taken into account when calculating the sample size**		
This issues was stated in the protocol paper of the study.		
**7b) CONSORT: When applicable, explanation of any interim analyses and stopping guidelines**		
Stated in the Data and Measurements and statistics section.		
**8a) CONSORT: Method used to generate the random allocation sequence**		
This issues was stated in the protocol paper of the study. https://doi.org/10.1186/s12889-019-6915-x		
**8b) CONSORT: Type of randomisation; details of any restriction (such as blocking and block size)**		
"The parents had to be literate in Norwegian.<br>Of the 404 recruited parents, 298 (73.8%) filled in more than<br>half of the baseline questionnaire which was the minimum<br>requirement to be randomized into either the control or<br>intervention group (n=148)."		
**9) CONSORT: Mechanism used to implement the random allocation sequence (such as sequentially numbered containers), describing any steps taken to conceal the sequence until interventions were assigned**		
This issues were stated in the protocol paper of the study. https://doi.org/10.1186/s12889-019-6915-x		
**10) CONSORT: Who generated the random allocation sequence, who enrolled participants, and who assigned participants to interventions**		
This issues were stated in the protocol paper of the study. https://doi.org/10.1186/s12889-019-6915-x		
**11a) CONSORT: Blinding - If done, who was blinded after assignment to interventions (for example, participants, care providers, those assessing outcomes) and how**		
**11a-i) Specify who was blinded, and who wasn't**		
NA		
**11a-ii) Discuss e.g., whether participants knew which intervention was the "intervention of interest" and which one was the "comparator"**		
NA		
**11b) CONSORT: If relevant, description of the similarity of interventions**		
NA		
**12a) CONSORT: Statistical methods used to compare groups for primary and secondary outcomes**		
"Means with standard deviations for continuous variables and<br>frequencies and percentages for categorical variables were<br>reported. The chi-square tests were used to test potential<br>differences in the perceived value of the intervention between<br>the 2 education groups and according to the number of children<br>in the household. Independent sample t tests were used to test<br>potential group differences for continuous variables."		
**12a-i) Imputation techniques to deal with attrition / missing values**		
Listed in the result section.		
**12b) CONSORT: Methods for additional analyses, such as subgroup analyses and adjusted analyses**		
We listed subgroup analyses.		
RESULTS		
**13a) CONSORT: For each group, the numbers of participants who were randomly assigned, received intended treatment, and were analysed for the primary outcome**		
NA (We use only data from the intervetion group, but the numbers are listed in the method section).		
**13b) CONSORT: For each group, losses and exclusions after randomisation, together with reasons**		
"In the intervention group, 93/148 (62.8%) participants answered<br>at least parts of the questionnaire at follow-up 1. However, only<br>83/148 (56.1%) participants answered the last questions in the<br>questionnaire that concerned the website use".		
**13b-i) Attrition diagram**		
Se table 2		
**14a) CONSORT: Dates defining the periods of recruitment and follow-up**		
This is listed in the study design section.		
**14a-i) Indicate if critical "secular events" fell into the study period**		
NA		
**14b) CONSORT: Why the trial ended or was stopped (early)**		
NA		
**15) CONSORT: A table showing baseline demographic and clinical characteristics for each group**		
Se table 1		
**15-i) Report demographics associated with digital divide issues**		
Se table 1		
**16a) CONSORT: For each group, number of participants (denominator) included in each analysis and whether the analysis was by original assigned groups**		
**16-i) Report multiple "denominators" and provide definitions**		
The the restult swection.		
**16-ii) Primary analysis should be intent-to-treat**		
Se table 2: "One participant got access to the intervention but decided to quit. Two did not get access to the intervention mistakenly. These 3 are included in the reported numbers."		
**17a) CONSORT: For each primary and secondary outcome, results for each group, and the estimated effect size and its precision (such as 95% confidence interval)**		
"Statistical significance was set to the P≤.05 level".		
**17a-i) Presentation of process outcomes such as metrics of use and intensity of use**		

See the result section.		
**17b) CONSORT: For binary outcomes, presentation of both absolute and relative effect sizes is recommended**		
NA		
**18) CONSORT: Results of any other analyses performed, including subgroup analyses and adjusted analyses, distinguishing pre-specified from exploratory**		
NA		
**18-i) Subgroup analysis of comparing only users**		
NA		
**19) CONSORT: All important harms or unintended effects in each group**		
NA		
**19-i) Include privacy breaches, technical problems**		
Not listed in this paper. See the protocol paper. https://doi.org/10.1186/s12889-019-6915-x		
**19-ii) Include qualitative feedback from participants or observations from staff/researchers**		
NA		
DISCUSSION		
**20) CONSORT: Trial limitations, addressing sources of potential bias, imprecision, multiplicity of analyses**		
**20-i) Typical limitations in ehealth trials**		
Se the strengths and limitaion section.		
**21) CONSORT: Generalisability (external validity, applicability) of the trial findings**		
**21-i) Generalizability to other populations**		
We higlight the biased sample of this intervention (highly educated mothers).		
**21-ii) Discuss if there were elements in the RCT that would be different in a routine application setting**		
NA		
**22) CONSORT: Interpretation consistent with results, balancing benefits and harms, and considering other relevant evidence**		
**22-i) Restate study questions and summarize the answers suggested by the data, starting with primary outcomes and process outcomes (use)**		
"More than 86.5% (128/148) of parents in the intervention group<br>visited the website and most of them found the website useful,<br>especially the modules and the recipes. The website content,<br>texts, and interface were highly valued by most parents, which<br>may have influenced parental engagement on the website.<br>Besides, most parents in the intervention group found the content<br>applicable to their child's age."		
**22-ii) Highlight unanswered new questions, suggest future research**		
IN the conclusion section: "Developing eHealth interventions and may inform future<br>interventions to take particular care in matching intervention<br>content to different educational and socioeconomic groups'<br>needs."		
Other information		
**23) CONSORT: Registration number and name of trial registry**		
"The study was approved by the Norwegian<br>Centre for Research Data on June 08, 2016 (reference number<br>48643)."		
**24) CONSORT: Where the full trial protocol can be accessed, if available**		
https://doi.org/10.1186/s12889-019-6915-x		
**25) CONSORT: Sources of funding and other support (such as supply of drugs), role of funders**		
"This study is funded by the University of Agder. The financial contributor was not involved in designing the study, collection, analyses, and interpretation of data or in writing the manuscript."		
**X26-i) Comment on ethics committee approval**		
"The study was approved by the Norwegian<br>Centre for Research Data on June 08, 2016 (reference number<br>48643)"		
**x26-ii) Outline informed consent procedures**		
Informed consent from parents was obtained when they<br>signed in online for participation.		
**X26-iii) Safety and security procedures**		
This issues were stated in the protocol paper of the study. https://doi.org/10.1186/s12889-019-6915-x		
**X27-i) State the relation of the study team towards the system being evaluated**		
This study is funded by the University of Agder. The financial contributor was not involved in designing the study, collection, analyses, and interpretation of data or in writing the manuscript.		
